# Differences in macular structure and microvasculature between high myopia and non-high myopia

**DOI:** 10.3389/fmed.2025.1645015

**Published:** 2025-08-26

**Authors:** Qin Chen, Keyao Song, Qing Cun, Wenyan Yang, Yijin Tao, Hua Zhong, Shitou Lv, Chaoxu Qian

**Affiliations:** ^1^Department of Ophthalmology, The First Affiliated Hospital of Nanjing Medical University, Nanjing, China; ^2^Department of Ophthalmology, The First Affiliated Hospital of Kunming Medical University, Kunming, China; ^3^Department of Ophthalmology, The People’s Hospital of Mengzi, Mengzi, China; ^4^Aier School of Ophthalmology, Central South University, Changsha, China; ^5^Aier Eye Hospital Group, Shanghai Aier Eye Hospital, Shanghai, China

**Keywords:** myopia, coherence tomography angiography, macular microvasculature, macular thickness, foveal avascular zone

## Abstract

**Purpose:**

To investigate the retinal and choroidal morphological and microvascular changes in myopic eyes using optical coherence tomography angiography (OCTA).

**Methods:**

In this retrospective, cross-sectional study, 142 eyes from 84 myopic patients were categorized into two groups based on spherical equivalent (SE): high myopia (HM; SE ≤ −6.0 D, 72 eyes) and non-high myopia (NHM; −6.0 D < SE ≤ −0.5 D, 70 eyes) groups. OCTA-derived parameters, including foveal retinal thickness (RT), choroidal thickness (CT), superficial retinal vessel density (SRVD) and deep retinal vessel density (DRVD), choriocapillaris density (CCD), and foveal avascular zone (FAZ) area and perimeter, were analyzed.

**Results:**

The mean SE was −10.16 ± 3.55 D in the HM group and −3.86 ± 1.60 D in the NHM group. RT measurements revealed substantial increases in HM eyes, including total foveal (276.4 ± 36.1 μm vs. 250.1 ± 23.3 μm; *p* < 0.01), parafoveal (348.0 ± 23.0 μm vs. 329.7 ± 20.8 μm; *p* < 0.01), and perifoveal regions (304.9 ± 25.1 μm vs. 290.6 ± 15.5 μm; *p* < 0.01). Retinal vascular analysis showed reduced SRVD in the fovea with borderline significance (14.9 ± 7.1% vs. 17.9 ± 7.4%; *p* = 0.05), while DRVD showed a non-significant reduction (31.4 ± 10.1% vs. 37.8 ± 24.0%; *p* > 0.05). HM eyes exhibited significant choroidal thinning (169.2 ± 74.0 μm vs. 222.4 ± 70.7 μm; *p* < 0.01) and decreased CCD (55.7 ± 3.1% vs. 58.0 ± 2.6%; *p* < 0.01). The FAZ displayed a larger area (0.4 ± 0.4 mm^2^ vs. 0.3 ± 0.1 mm^2^; *p* = 0.07) and perimeter (2.5 ± 1.1 mm vs. 2.1 ± 0.4 mm; *p* < 0.05) in HM subjects.

**Conclusion:**

High myopia is associated with distinct retinal and choroidal alterations, including increased RT, reduced CT and CCD, and enlarged FAZ, suggesting progressive microvascular and structural remodeling with myopia severity.

## Introduction

Myopia is a global public health concern, with projections suggesting that it will affect nearly half of the world’s population by 2050, of whom 9.8% will have high myopia (HM) (1). HM is a significant risk factor for vision-threatening complications due to its association with retinal and choroidal pathologies, including myopic maculopathy, choroidal atrophy, and retinal detachment (2). Despite its clinical importance, the morphological and microvascular changes in the retina and choroid of myopic eyes remain poorly understood, with conflicting evidence in the literature.

Morphological changes within the retina and choroid are significantly linked to high myopia (3). Structural changes in the posterior segment of the eye caused by HM, such as posterior staphyloma, retinal atrophy, and myopic maculopathy, contribute to irreversible visual impairment (4). While retinal thickness has been reported to vary with myopia severity, the relationship between macular intraretinal layer thickness, microvascular alterations, and functional visual outcomes remains unclear (5). The choroid, a critical vascular layer supplying the outer retina, is increasingly recognized as a key player in the regulation of myopia development and progression (6). Historically, technical limitations hindered detailed choroidal assessment, but recent advances in imaging have revealed its role in myopia-associated changes, though findings remain inconsistent.

Optical coherence tomography angiography (OCTA) has revolutionized non-invasive imaging by enabling the simultaneous evaluation of retinal and choroidal morphology and microvasculature. Prior studies using OCTA reported conflicting results: most observed reduced retinal vessel density and choriocapillaris density (CCD) among patient in the HM group compared to those in the non-high myopia (NHM) group (5, 7–14), while others found no significant differences in retinal vessel density (15, 16) or CCD (17). These discrepancies may arise from variations in study design, sample characteristics, or imaging protocols, underscoring the need for further investigation.

Elucidating the structural and microvascular changes in myopia could provide insights into strategies for mitigating its progression and complications. In this study, we quantitatively compared macular retinal/choroidal thickness, vessel density, and foveal avascular zone (FAZ) parameters between HM and NHM patients to clarify these relationships.

## Methods

This retrospective, cross-sectional study included patients with high myopia, from the First Affiliated Hospital of Kunming Medical University and the First Affiliated Hospital of Nanjing Medical University. Age- and gender-matched patients with mild to moderate myopia were enrolled as controls during the same period. The study protocol was approved by the Ethics Committees of both institutions and complied with the Declaration of Helsinki. Written informed consent was obtained from all participants.

Study eyes were divided into two groups based on spherical equivalents (SE), calculated as the spherical dioptric power plus one-half of the cylindrical dioptric power. Eyes with SE ≤ −6.00 diopters (D) were categorized as HM, while eyes with SE > −6.00 D and ≤ − 0.50 D were designated as NHM, which was also considered the control group. The exclusion criteria included astigmatism greater than 2.00 D, intraocular pressure (IOP) exceeding 21 mmHg, history of ocular trauma, intraocular surgery, retinal disease, any ocular or systemic disorders that might affect ocular circulation, and participants unable to complete the OCTA examination.

All subjects underwent comprehensive ocular examinations including best-corrected visual acuity (BCVA), slit-lamp examination, fundoscopy, refractive status assessed by an automatic refractometer (KR-8900; Topcon, Tokyo, Japan), non-contact tonometry (CT-1P; Topcon, Tokyo, Japan) for IOP, fundus photography (Visucam 200; Carl Zeiss Meditec AG, Germany) after cycloplegia, and axial length (AL) measurement via optical biometry (IOL Master; Carl Zeiss Meditec, Jena, Germany).

After pupillary dilation, OCTA scans of the macular regions were obtained using a commercially available RTVue XR OCT (Optovue, Fremont, California, USA) with the HD Angio retina mode (6 × 6 mm). The technique of OCTA has been described in detail previously (5, 7–13, 15–17). Automatic segmentation by the inbuilt software generated en face projection images of superficial retinal vessel density (SRVD), deep retinal vessel density (DRVD), and choriocapillaris density (CCD). Image quality control excluded scans with signal strength index less than six, segmentation errors, motion, and decentration artifacts. Magnification effects due to axial length were corrected using Littman’s formula and Bennett’s algorithm (18). Briefly, according to Littman’s formula, the true size *t* of a retinal feature on an OCTA image can be expressed as *t* = *p* × *q* × *s*, where *p* is a constant value of 3.382, *q* is the magnification factor related to the eye, and *s* is the measurement. The factor *q* can be determined by Bennett’s formula: *q* = 0.01306 × (*x* − 1.82), where *x* is the axial length.

The FAZ metrics, including area (mm^2^) and perimeter (mm), were automatically generated by the built-in software. Similarly, the superficial and deep retinal vessel densities and retinal thickness measurements for the foveal, parafoveal, and perifoveal regions were automatically calculated by the system’s proprietary algorithms. Choroidal thickness was manually measured on enhanced HD line chorioretinal B-scans, with measurements taken from Bruch’s membrane to the choroid-sclera interface at the foveal center. All manual measurements were performed by an experienced ophthalmologist (K. S.) and verified by a senior ophthalmologist (H. Z.) to ensure accuracy.

**Figure 1:**
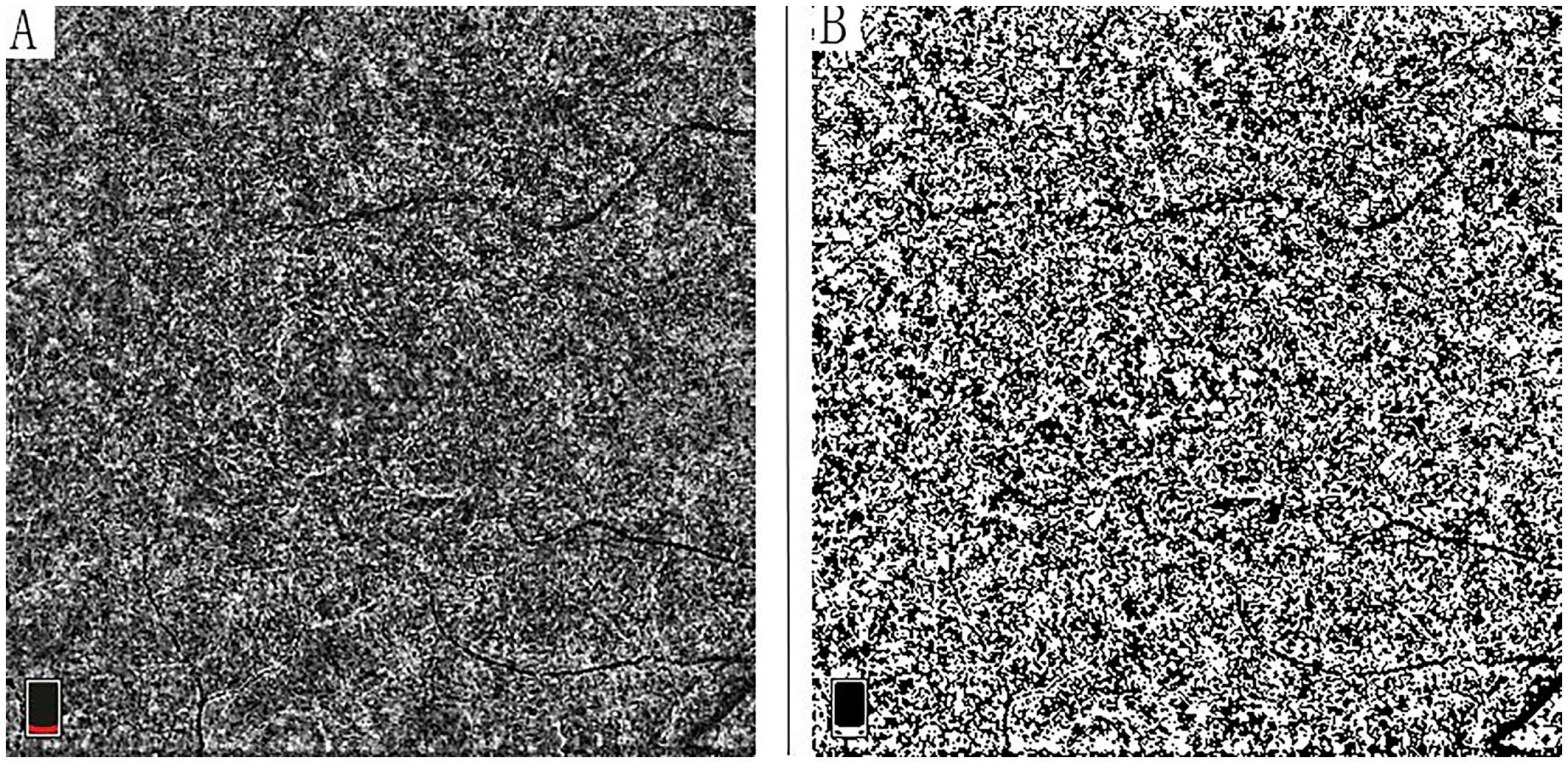
Quantitative analysis of choriocapillaris vessel density using OCT angiography. **(A)** Original 6 × 6 mm en face OCTA projection image of the choriocapillaris layer. **(B)** Corresponding binarized image generated using the Phansalkar method in ImageJ software (version 1.53; National Institutes of Health), demonstrating the flow signal used for choriocapillaris density (CCD) calculation. CCD was quantified as 1.0 minus flow deficit density using the analyze particles function.

For CCD analysis, OCTA images were processed using ImageJ software (version 1.53, National Institutes of Health, Bethesda, MD, USA). The 6 × 6 mm images were output from the instrument and were analyzed using the Phansalkar method with a default window radius of 15 pixels. The images were first converted to 8-bit format and subsequently binarized using the Phansalkar method (19). CCD, calculated as 1.0 minus the flow deficit density, was performed using the “Analyze Particles” command (Figure 1) (13, 19).

## Statistical analysis

Given the limitations of previous evidence, we applied the preliminary data to estimate the sample size. In our preliminary data analysis, the CT exhibited the highest variability among the measured parameters. The mean CT values were 210 μm in the NHM group and 180 μm in the HM group, with a comparable SD of 60 μm in both groups. With the power set at 80% and *α* at 0.05, the estimated sample size was 64 for each group, and the total sample size was 128. In our study, the eyes enrolled for analysis were 172, exceeding the calculated minimum. Data were analyzed using the Statistical Package for the Social Sciences (SPSS), version 25.0 (SPSS, Inc., IBM Corporation, Chicago, IL, USA). The normality of data distribution was evaluated using the Shapiro–Wilk test. Continuous variables with a normal distribution were compared between the HM and NHM groups using linear mixed-effect models, after adjusting for the clustering effect between eyes within the same person by controlling for the covariates. The Pearson correlation analysis was conducted to examine the relationships between SE and each parameter. A *p*-value of less than 0.05 was considered statistically significant.

## Results

### Demographic and clinical characteristics

A total of 142 eyes from 84 myopic patients were included in this study. The mean age of the patients was 33.6 ± 13.1 years. The demographic and clinical information of the study population is presented in Table 1. The eyes were stratified into two groups based on SE: the high myopia group (HM, SE ≤ −6.0 D, 72 eyes) and the non-high myopia group (NHM, −6.0 D < SE ≤ −0.5 D, 70 eyes). The HM group demonstrated significantly greater AL and worse BCVA compared to the NHM group (*p* < 0.01).

**Table 1:** Demographic and clinical characteristics stratified by spherical equivalent.

Characteristic	Total	NHM	HM	*p* value
Participants, *N*	84	40	44	
Eyes, *n*	142	70	72	
Age, years	33.6 ± 13.1	31.6 ± 10.5	35.6 ± 14.9	0.09
Female, %	93 (65.5%)	46 (65.7%)	47 (65.3%)	0.80
SE, D	−7.07 ± 4.16	−3.86 ± 1.60	−10.16 ± 3.55	< 0.01
AL, mm	26.14 ± 1.87	24.86 ± 1.21	27.39 ± 1.53	< 0.01
BCVA (logMAR)	0.07 ± 0.13	0	0.14 ± 0.15	< 0.01

Data are presented as mean ± standard deviation (SD) or as number and percentage. NHM, non-high myopia (−6.0 D < SE ≤ − 0.5 D); HM, high myopia (SE ≤ − 6.0 D); SE, spherical equivalent; AL, axial length; BCVA, best correct visual acuity.

### Retinal thickness and vessel density between HM and NHM eyes

**Figure 2:**
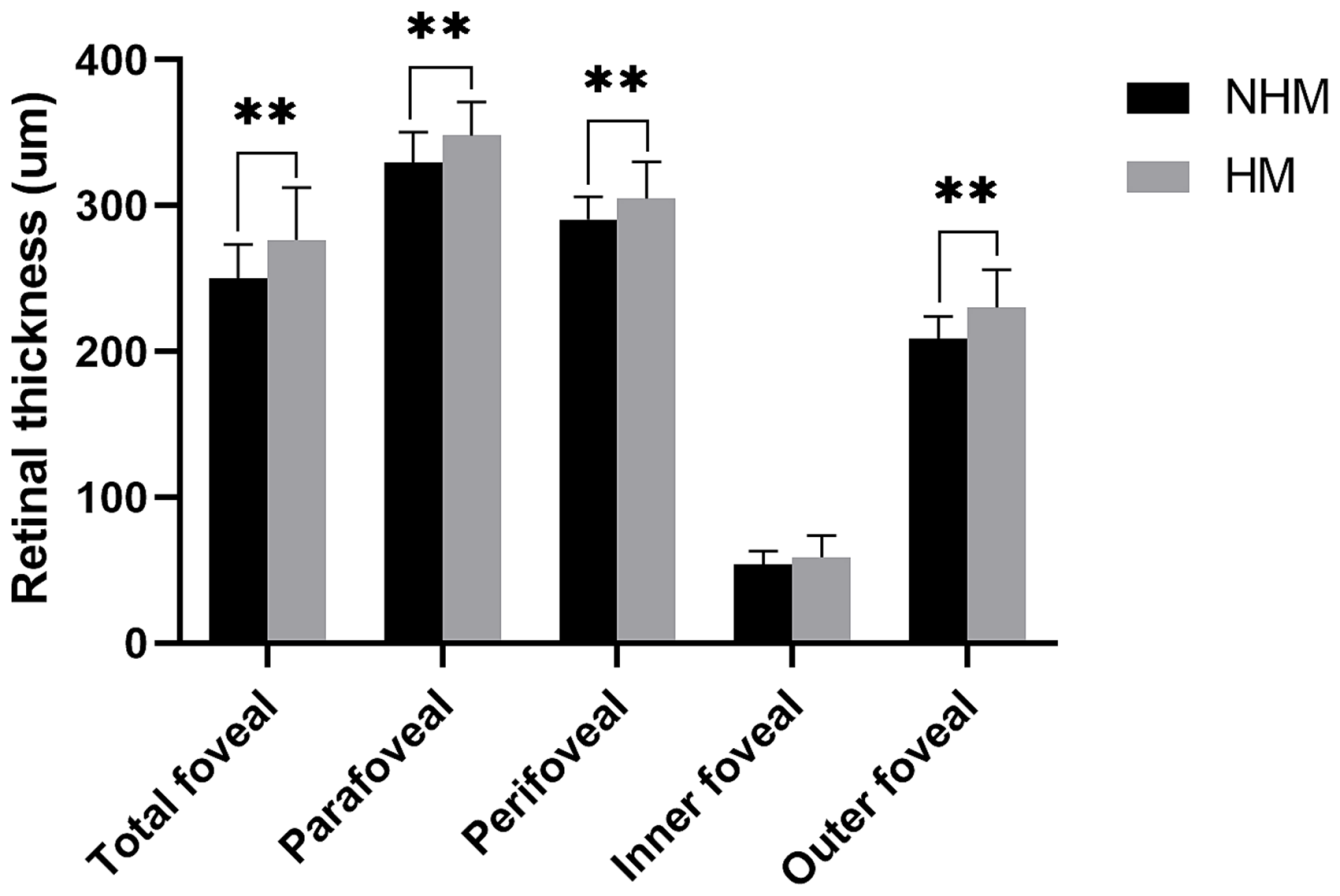
The histogram plot of retinal thickness. Comparative analysis of retinal thickness profiles between the high myopia (HM) and non-high myopia (NHM) groups. Histogram illustrates thickness measurements (μm) across different macular regions, demonstrating significant differences between the two groups. * *p* < 0.05, ** *p* < 0.01.

The macular RT measurements were consistently greater in the HM group compared to NHM controls, with significant differences observed in the total foveal (276.4 ± 36.1 μm vs. 250.1 ± 23.3 μm; *p* < 0.01), parafoveal (348.0 ± 23.0 μm vs. 329.7 ± 20.8 μm; *p* < 0.01), and perifoveal (304.9 ± 25.1 μm vs. 290.6 ± 15.5 μm; *p* < 0.01) regions. Layer-specific analysis revealed that the outer retinal layers were significantly thicker in the HM group compared to the NHM eyes (230.3 ± 25.8 μm vs. 208.7 ± 15.4 μm; *p* < 0.01). While the inner retinal layers showed a similar trend toward increased thickness in HM eyes (58.7 ± 15.0 μm vs. 53.9 ± 9.2 μm), this difference did not reach statistical significance (*p* = 0.09) (Figure 2).

**Figure 3:**
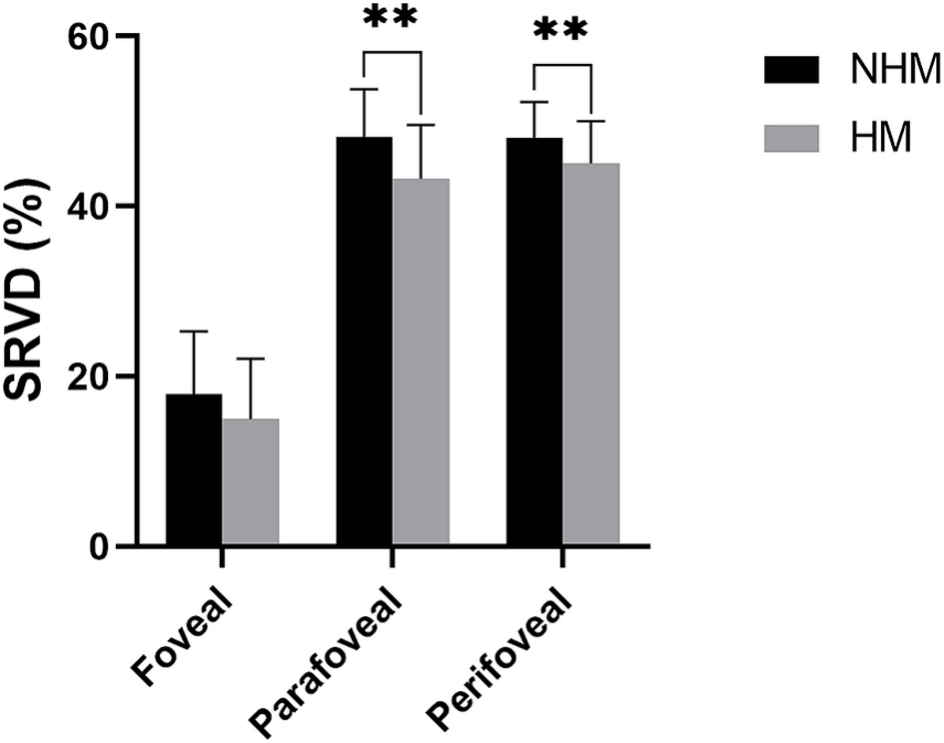
The histogram plot of superficial retinal vessel density (SRVD). Comparative analysis of SRVD between the high myopia (HM) and non-high myopia (NHM) groups. Histogram displays SRVD (%) measurements across different macular regions, demonstrating reductions in HM eyes versus NHM controls. * *p* < 0.05, ** *p* < 0.01.

Quantitative analysis revealed significantly reduced SRVD in the HM group compared to the NHM control group across all macular regions. The foveal SRVD demonstrated a borderline significant reduction in HM eyes (14.9 ± 7.1% vs. 17.9 ± 7.4%; *p* = 0.05), while more pronounced decreases were observed in both the parafovea (43.2 ± 6.4% vs. 48.1 ± 5.6%; *p* < 0.01) and perifovea (45.0 ± 5.0% vs. 48.1 ± 4.2%; *p* < 0.01) regions (Figure 3).

**Figure 4:**
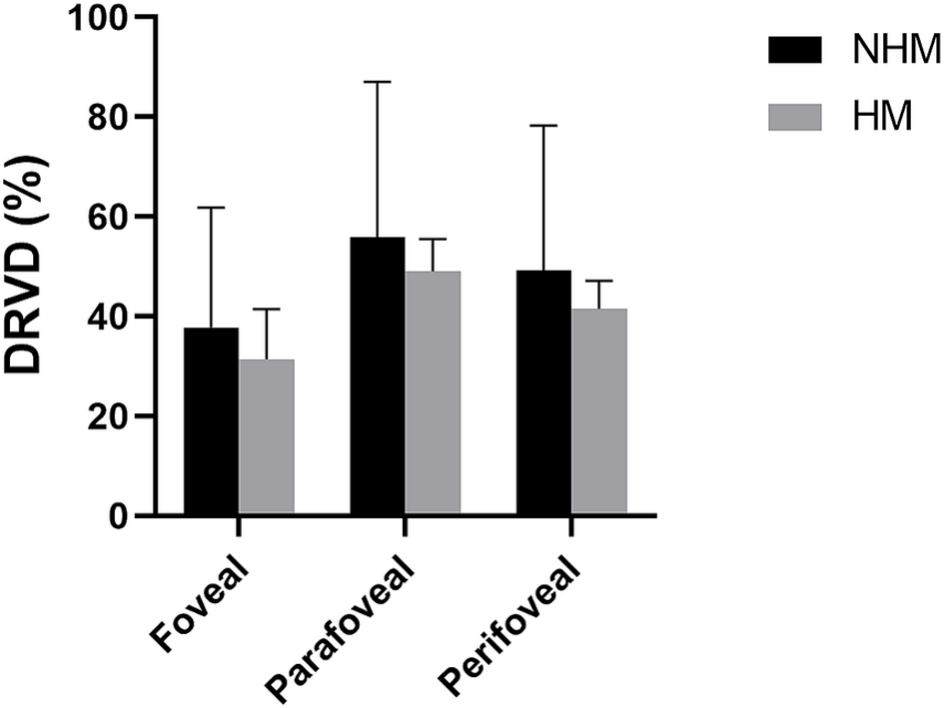
The histogram plot of deep retinal vessel density (DRVD). Comparative analysis of DRVD profiles between the high myopia (HM) and non-high myopia (NHM) groups. Histogram illustrates regional DRVD (%) measurements, showing reductions in HM eyes versus NHM controls, although observed difference did not reach statistical significance.

Similarly, DRVD in the HM group was reduced in the fovea (31.4 ± 10.1%), parafoveal (49.1 ± 6.4%), and perifovea (41.5 ± 5.6%) regions compared to the NHM control group (fovea: 37.8 ± 24.0%; parafoveal: 55.8 ± 31.2%, and perifovea: 49.1 ± 29.1%). These differences did not reach statistical significance, *p* > 0.05 (Figure 4).

### Choroidal thickness and choriocapillaris density between HM and NHM eyes

**Figure 5:**
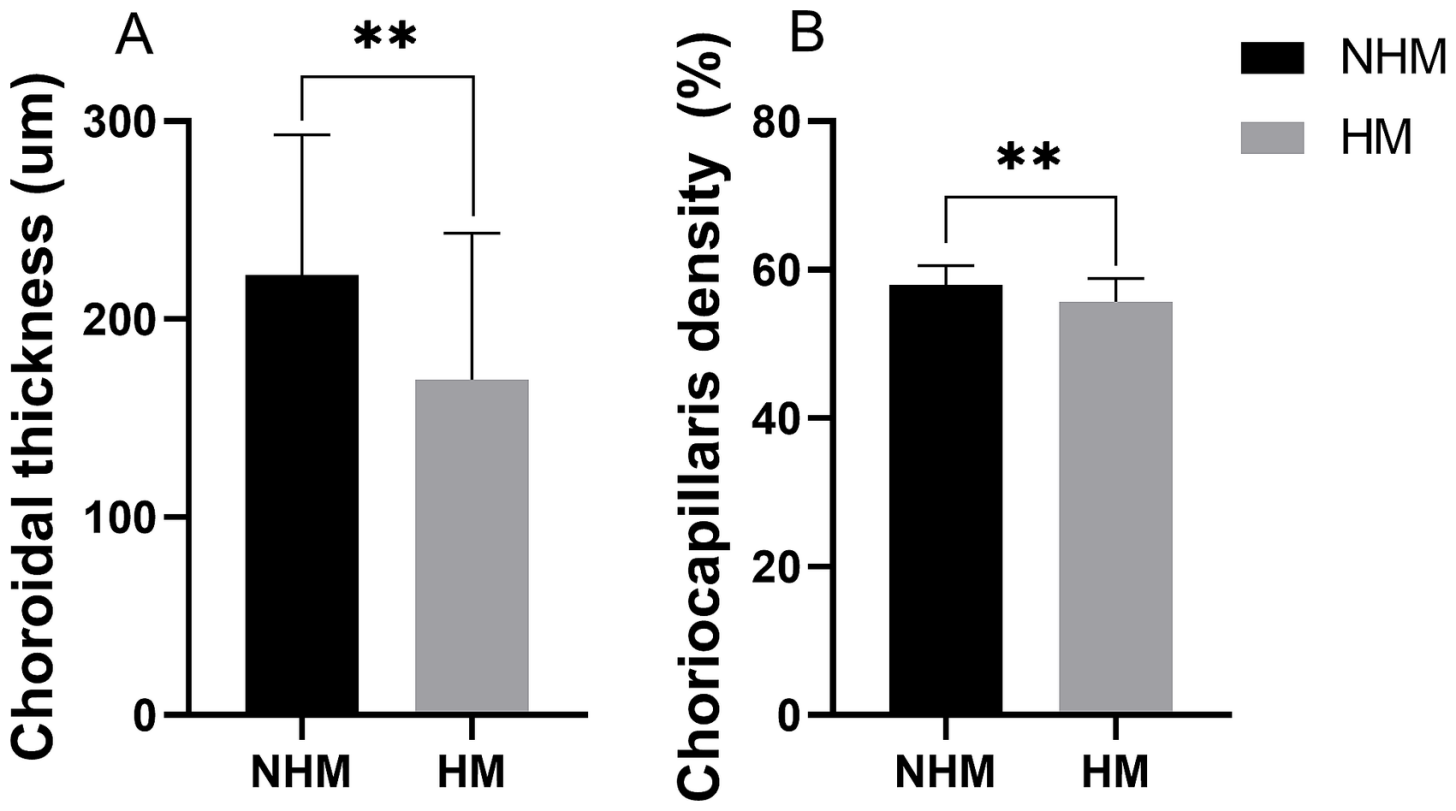
The histogram plot of choroid thickness (CT) and choriocapillaris density (CCD). Quantitative comparison of choroidal parameters between the high myopia (HM) and non-high myopia (NHM) groups. **(A)** CT measurements show significant thinning in HM eyes. **(B)** CCD demonstrating reduced vascular perfusion in the HM group. * *p* < 0.05, ** *p* < 0.01.

A total of 142 B-scans were taken and used for analysis. The total average CT was 195.5 ± 76.9 μm. The CT in the HM group (169.2 ± 74.0 μm) demonstrated significant thinning compared to the NHM control group (222.4 ± 70.7 μm; *p* < 0.01, Figure 5A). The CCD in the HM group (55.7 ± 3.1%) was significantly lower than that in the NHM control group (58.0 ± 2.6%, *p* < 0.01, Figure 5B).

### Foveal avascular zone characteristics between HM and NHM eyes

The FAZ metrics exhibited enlargement of eyes in the HM group compared to eyes in the NHM group, with both area (0.4 ± 0.4 mm^2^ vs. 0.3 ± 0.1 mm^2^; *p* = 0.07) and perimeter (2.5 ± 1.1 mm vs. 2.1 ± 0.4 mm; *p* < 0.05) demonstrating measurable increases (Table 2).

**Table 2:** The area and perimeter of FAZ in high myopia and non-high myopia eyes.

FAZ	Total	HM	NHM	*P* value
Area, mm^2^	0.3 ± 0.3	0.4 ± 0.4	0.3 ± 0.1	0.07
Perimeter, mm	2.3 ± 0.8	2.5 ± 1.1	2.1 ± 0.4	0.01

Data presented as mean ± SD. FAZ, the foveal avascular zone. HM, high myopia. NHM, non-high myopia.

### The correlation between SE and each parameter

**Figure 6:**
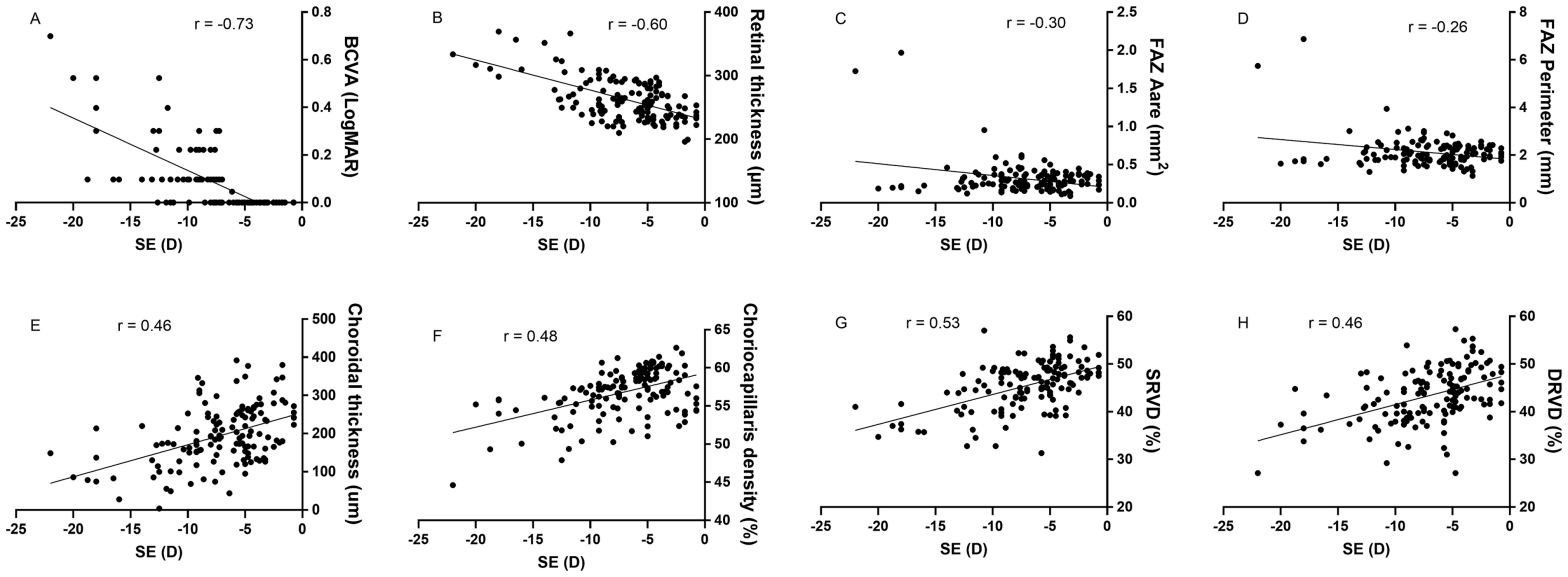
The correlation between spherical equivalent (SE) and ocular parameters. SE exhibits negative correlations with best correct visual acuity (BCVA), retinal thickness, and foveal avascular zone (FAZ) area and perimeter, while showing positive correlations with choroidal thickness, choriocapillaris density, superficial retinal vessel density (SRVD) and deep retinal vessel density (DRVD). All correlations are statistically significant (*p* < 0.01).

For the total of 142 myopic eyes, SE showed significant negative correlations with BCVA (*r* = −0.73, *p* < 0.01), RT (*r* = −0.60, *p* < 0.01), FAZ area (*r* = −0.30, *p* < 0.01), and FAZ perimeter (*r* = −0.26, *p* < 0.01). Conversely, SE demonstrated significant positive correlations with CT (*r* = 0.46, *p* < 0.01), CCD (*r* = 0.48, *p* < 0.01), SRVD (*r* = 0.53, *p* < 0.01), and DRVD (*r* = 0.46, *p* < 0.01) (Figure 6).

## Discussion

Our study reveals significant microvascular and structural alterations distinguishing HM eyes from NHM eyes. Specifically, HM eyes exhibited reduced retinal vessel density in both superficial and deep vascular plexuses, decreased CCD, and thinner subfoveal CT, while demonstrating increased subfoveal RT and enlarged FAZ dimensions compared to NHM controls. The observed patterns of retinal and choroidal vascular attenuation, coupled with FAZ enlargement, suggest that microvascular insufficiency may represent both a biomarker and a potential mechanistic factor in high myopia development.

Previous investigations have consistently demonstrated the critical role of choroidal changes in myopia development. As the primary vascular supply for the outer retina, the choroid exhibits remarkable plasticity in response to refractive demands (6). Animal studies first established this relationship in the 1990s, showing choroidal thickness modulation in response to optical defocus in avian models (20). Subsequent human studies by Read et al. (21) confirmed similar choroidal adaptations, supporting the choroid’s active role in emmetropization and myopia development.

The choriocapillaris, the innermost layer of the choroidal vasculature, consists of a small diameter and fenestrated capillaries located beneath Bruch’s membrane. Historically, technical limitations in imaging modalities constrained our understanding of choriocapillaris function. While indocyanine green angiography (ICG) has served as a primary choroidal imaging technique, its invasive nature and its inability to specifically isolate the choriocapillaris layer have restricted its utility. The advent of OCTA has revolutionized this field by enabling non-invasive, high-resolution visualization of choroidal vasculatures. Recent advances in image processing, particularly binarization techniques, have facilitated quantitative assessment of choroidal vascular luminal area, thereby extending our analytical capabilities beyond simple thickness measurements to explore potential correlations between microvascular alterations and myopic progression (19, 22).

Our findings demonstrate significant reductions in both choroidal thickness and CCD in HM eyes compared to NHM controls, aligning with established literature documenting an inverse relationship between choroidal thickness and myopia progression (23). This choroidal attenuation appears to be progressive, with studies showing decreasing thickness correlating with both myopia onset and severity (24). Current evidence consistently identifies choroidal thinning as a hallmark structural alteration in myopia (23), with particular significance in HM, where marked thinning has been established as an early and characteristic change (6). Wu et al. developed an artificial intelligence-based architecture for choroid segmentation, offering a simple approach to revealing morphological changes in myopia (25). Recent investigations utilizing OCTA quantification of choriocapillaris flow deficit percentage (CC FD%)—representing regions of subthreshold or impaired blood flow—have reported elevated CC FD% in HM eyes (10, 14). OCTA also revealed significant reductions in both choroidal thickness and circulation in eyes with greater myopic refraction among anisomyopic subjects (26). Supporting evidence from chick myopia models revealed that choriocapillaris alterations are followed by a decrease in choroidal blood flow (27), suggesting that choroidal thinning might be associated with decreased blood flow (23, 24). While Yang et al. attributed these vascular changes primarily to vessel diameter constriction (28), other studies suggested that these changes were associated with reductions in vascular density, stromal components, and choriocapillaris thinning itself (10, 29).

Our study revealed significantly greater retinal thickness in the foveal, parafoveal, and perifoveal regions of HM patients compared to NHM controls after ocular magnification adjustment (all *p* < 0.05). These findings corroborate evidence from existing literature demonstrating retinal thickening associated with higher myopic degrees (30–32). Kim et al. specifically documented increased thickness across all individual retinal layers throughout the macular region in HM versus NHM subjects (30). Population-level evidence from Duan et al.’s study of 6,830 Chinese adults further supports this relationship, showing positive correlations between axial length and macular thickness (31). Longitudinal data from pediatric cohorts additionally confirm progressive macular thickening accompanying myopia progression (32). While generally consistent with these reports, our results partially contrast with the findings by Liu et al. (33), who found that some retinal layers increased but not the total mean retinal thickness, with a higher myopia. Another study found that the RT decreased with myopia progression (34). However, this study utilized 12 × 12 mm imaging without accounting for magnification effects, potentially confounding the results. The relationship between RT and myopia remains controversial. Our study demonstrates that high myopia tends to increase macular RT. The underlying mechanisms for this association remain unclear. High myopia may be associated with more segmentation errors because long AL induced morphological changes. To minimize these segmentation artefacts, all automated segmentation lines by the instrument were manually verified by two experienced ophthalmologists, with special attention given to high myopic eyes with staphyloma. Magnification correction plays an important role in calculating the actual measurements. Kang et al. (35) demonstrated that scan circle magnification exceeds 5% in eyes with myopia exceeding −4.00 D and recommended that ocular magnification adjustment should be considered when myopia exceeds −4.00 D. The observed retinal thickening in HM may be explained by the differential axial elongation effects across retinal regions—while equatorial and per-equatorial areas demonstrate marked changes, foveal thickness remains relatively stable (36), supporting the concept of macular thickness independence from axial length variations (4).

Our study additionally demonstrated significantly larger FAZ dimensions (both area and perimeter) in HM patients compared to NHM controls, aligning with previous reports of FAZ enlargement in high myopia (37). These findings are substantiated by a dedicated FAZ analysis study involving 106 healthy myopic participants stratified by axial length quartiles, where the upper quartile (HM group) exhibited significantly greater FAZ parameters than the lower quartile (NHM group) when measured by OCTA (37). Further supporting evidence has been gathered from studies documenting concurrent FAZ area enlargement and macular VD reduction (38), suggesting that FAZ characteristics may serve as an indirect indicator of retinal perfusion status. The observed FAZ changes may result from mechanical vascular trunk displacement during axial elongation, potentially leading to compromised blood flow. However, our results contrast with those of Zivkovic et al. (39), who reported no significant FAZ differences between myopic and control eyes. However, they obtained the measurements without adjusting the magnification.

Several limitations should be acknowledged in the current investigation. First, the retrospective cross-sectional design and relatively small sample size constrain our ability to establish causal relationships between observed microvascular and structural changes in myopic eyes. Future prospective studies with larger cohorts are warranted to validate these findings and elucidate potential causal mechanisms. Second, methodological variations in imaging protocols may affect the comparability of the results. While our analysis utilized 6 × 6 mm en face OCTA scans, other studies have employed different field sizes (e.g., 3 × 3 mm, 12 × 12 mm central 1-mm macular zone) (12, 34, 40). These technical differences in scan dimensions could influence vessel density measurements and limit direct comparisons across studies. Third, our study did not specifically assess intra-or inter-grader repeatability for CT. However, we implemented rigorous quality control protocols. All manual measurements were performed by an experienced ophthalmologist (K. S.) and subsequently verified by a senior ophthalmologist (H. Z.) to ensure measurement accuracy.

In summary, our OCTA-based investigation, incorporating appropriate magnification adjustment, revealed distinct microvascular and structural alterations in HM eyes compared to NHM controls. High myopia is associated with distinct retinal and choroidal alterations, including increased RT, reduced CT and CCD, and enlarged FAZ, suggesting progressive microvascular and structural remodeling with myopia severity. The identified patterns of vascular attenuation and structural remodeling provide valuable insights for developing targeted strategies to monitor and potentially mitigate myopia-related complications. However, future prospective studies with larger cohorts are required to validate these findings and elucidate the precise mechanisms underlying these observed changes.

## Data Availability

The raw data supporting the conclusions of this article will be made available by the authors, without undue reservation.
